# Sensitivity of quantum magnetic sensing

**DOI:** 10.1093/nsr/nwaf129

**Published:** 2025-04-04

**Authors:** Liwei Lei, Teng Wu, Hong Guo

**Affiliations:** State Key Laboratory of Photonics and Communications, School of Electronics, and Center for Quantum Information Technology, Peking University, China; State Key Laboratory of Photonics and Communications, School of Electronics, and Center for Quantum Information Technology, Peking University, China; State Key Laboratory of Photonics and Communications, School of Electronics, and Center for Quantum Information Technology, Peking University, China

## Abstract

Magnetometer, quantum or classical? Unveil the fundamental limit of magnetic field sensing.

The sensing of a magnetic field with ultra-high sensitivity can be achieved using quantum magnetometers, which are based on the quantum natures of micro-particles. These quantum natures, namely, discreteness and quantum coherences (including quantum entanglement), are dominant factors that equip quantum magnetometers with properties, e.g. ultra-high sensitivity, which are otherwise hardly achievable with classical sensors. Quantum magnetometers have found broad applications in fundamental physics, non-invasive biomedical diagnostics and remote sensing.

Quantum magnetometers refer to two typical cases: superconducting quantum interference devices based on superconducting loops containing Josephson junctions, and atomic magnetometers based on various spin species, ranging from an unpaired valence electron in alkali-metal atoms, excited metastable $^{4}$He atoms, and also a nucleus (proton and/or neutron) in water, $^{3}$He and Xe, as well as Hg and Yb atoms. The working mechanism of these two kinds of quantum magnetometers is tightly related to the manipulation of the state (ensemble) evolution of micro-particles in the presence of an ambient magnetic field. With the development of laser and superconducting technologies, both the state (ensemble) manipulation and readout become increasingly efficient, paving the way for quantum magnetometry to become the state-of-the-art solution for magnetic sensing nowadays. So far, the most sensitive quantum magnetometer is demonstrated to be capable of measuring a magnetic field at

the level of fT/$\sqrt{\text{Hz}}$, roughly one billionth of the geomagnetic field.

It is now necessary to examine some fundamental questions related to the ultra-high sensitivity of magnetometers. First, is there a *fundamental limit* for the capability of magnetometers? Second, to what degree can we conclude that the magnetometer is essentially *quantum*? Unraveling the fundamental principles may help to address these two questions.

Here, we take the atomic magnetometer as an example for discussion. Sensitivity, reflecting the smallest detectable variation in a magnetic field for a given time duration, is a critical performance metric for the atomic magnetometer, and is primarily constrained by technical and quantum noises. For ideal cases, i.e. if technical noise could be well controlled and suppressed, sensitivity of the atomic magnetometer is dominated by quantum noise. This provides a direct way to evaluate the quantumness of the magnetometer by comparing the measured noise with quantum noise limits. However, is it enough?

Two typical quantum noises are spin projection noise (SPN) and photon shot noise (PSN), both of which are commonly used to estimate the ultimate sensitivity of atomic magnetometers. Take SPN as an example: for an *N*-atom ensemble system with gyromagnetic ratio $\gamma$, given a total measurement duration *T* and a single measurement duration close to the relaxation time $\tau$, SPN is given by $\delta B_{\text{SPN}} \approx 1/(\gamma \sqrt{NT\tau })$[1]. It should be noted that the assumption for deriving SPN is that the atoms are uncorrelated (i.e. independently and identically distributed) and quantum noise in individual measurement is constrained only by the Heisenberg uncertainty principle. Therefore, using SPN and PSN to evaluate sensitivity has restrictions, and is inappropriate for evaluating the noises of correlated atom ensembles, and lacks ways for analyzing some sensing schemes.

Compared to SPN and PSN, quantum parameter estimation (QPE) provides a general framework for analyzing the sensitivity. By modeling the quantum sensing scheme as four steps—state preparation, encoding, readout and estimation—researchers utilize quantum Fisher information (QFI) to evaluate the sensitivity, with its ultimate limit given by the quantum Cramér-Rao bound (QCRB)[2]. In the case of uncorrelated initial states, the QCRB gives the standard quantum limit (SQL), which is consistent with SPN and PSN, as $\delta B_{\text{SQL}}\propto 1/\sqrt{N}$ [3]. Considering particle correlations, such as using squeezed or entangled states, the sensitivity can reach the Heisenberg limit (HL), where $\delta B_{\text{HL}}\propto 1/N$ [3]. In contrast, SPN and PSN cannot achieve the HL, as they assume no correlations among particles. Approaching the Heisenberg limit is closely linked to entanglement, meaning that QFI, which determines the best achievable measurement sensitivity, can also serve as a criterion for multi-partite entanglement [4]. QPE considers the limits of statistical estimations under an optimal quantum measurement scheme, highlighting the

fact that reaching the ultimate sensitivity requires perfectly transferring magnetic field parameter information. This approach is fundamentally constrained by the Heisenberg uncertainty principle and statistical estimation theory.

Numerous experiments have proved that the sensitivity limits of different kinds of magnetometers could be unified to a compact formula, i.e. the energy resolution limit (ERL) [5]. For volumetric sensors, including optically pumped magnetometers, the ERL is expressed as $(\delta B)^2 V T / 2\mu _0 \gtrsim \hbar$, where $(\delta B)^2$ represents the estimated variance of the magnetic field, *V* is the sensor volume, *T* is the measurement duration and $\mu _0$ is the vacuum permeability. The relation describes the product of the magnetic field energy within a finite volume and the measurement duration, bounded by $\hbar$. The sensitivity of magnetic field energy measurements depends on the lifetime of the quantum state, making the ERL a valuable metric to evaluate the quantumness of processes, such as biomagnetic sensing [6]. Researchers have recently proposed a first-principle derivation of the ERL from the

perspective of quantum thermodynamics and quantum speed limits: the quantum thermodynamic work accompanying the process of measurement is taken up by the magnetic field energy, and thus leads to magnetic field fluctuations [7]. Since the quantum thermodynamic work is derived under thermal equilibrium, and the physics of spin relaxation in correlated vapors remains poorly understood, we cannot make strong statements about the general validity of the ERL, and the potential for sensing with unconstrained energy resolution remains an open issue [7–9]. Up to now, a universally accepted derivation for the ERL is still lacking.

It should be noted that the sensitivity limits discussed above are all interconnected. SPN and PSN are practical cases of the SQL, applied to atomic spins and photons. Some researchers attempt to unify these two quantum noises in atomic magnetometers [10]. When the particles are uncorrelated, the ERL can be used to derive SPN [7]. Within the scheme of QPE, considering that a complete measurement cycle requires erasure of the memory information, a connection can be made between thermodynamic dissipation and QFI [11]. Some studies also give the relation between QFI and quantum speed limits [12]. Combining the first-principle derivation of energy resolution, it might be possible to establish a bridge between the QCRB and ERL.

To summarize, the fundamental sensitivity limit of magnetometers, whether evaluated by SPN/PSN, the QCRB or the ERL, is fundamentally confined by, say, the uncertainty principle, statistical estimation theory and thermodynamics of information (Fig. 1). Compared to directly using SPN and PSN for evaluating the sensitivity, QPE, which derives the QCRB for specific sensing protocols, is well developed and demonstrates broader applicability. In contrast to SPN, PSN and QPE, the ERL, which is independent of specific sensing protocols, seems to be more suitable to capture the essence of the sensing limit. These evaluation methods establish the fundamental limits of quantum magnetometers from different perspectives. Achieving these limits can serve as a critical criterion to determine whether a magnetometer operates at the quantum level. Understanding the relationships and underlying principles of these methods is essential for optimizing performance and evaluating their quantum characteristics.

**Figure 1.**
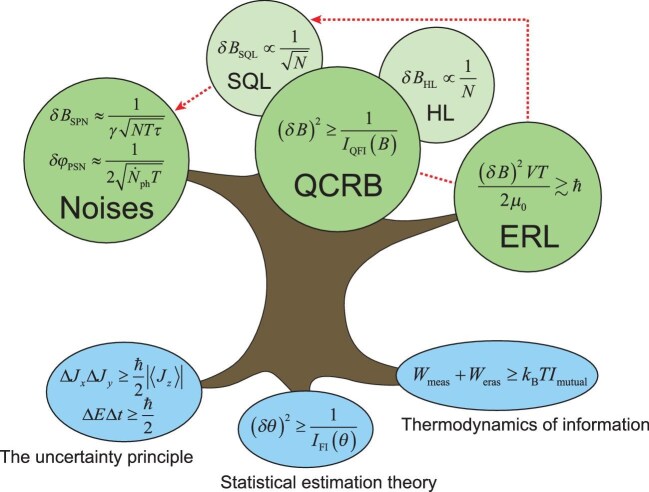
Three sensitivity limits of quantum magnetometers and their fundamental constraints. The tree diagram shows three perspectives for evaluating sensitivity limits (green leaves): noises, the QCRB (including the SQL and HL) and the ERL. These perspectives are interconnected (red dashed lines) and are constrained by fundamental principles (blue sources connected to the roots), such as the uncertainty principle, statistical estimation theory and thermodynamics of information.

## References

[1] Budker D, Romalis M. Nat Phys 2007; 3: 227–34. 10.1038/nphys566

[2] Braunstein SL, Caves CM. Phys Rev Lett 1994; 72: 3439. 10.1103/PhysRevLett.72.3439 10056200

[3] Giovannetti V, Lloyd S, Maccone L. Nat Photon 2011; 5: 222–9. 10.1038/nphoton.2011.35

[4] Hyllus P, Laskowski W, Krischek R et al. Phys Rev A 2012; 85: 022321. 10.1103/PhysRevA.85.022321

[5] Mitchell MW, Palacios Alvarez S. Rev Mod Phys 2020; 92: 021001. 10.1103/RevModPhys.92.021001

[6] Kominis IK, Gkoudinakis E. PRX Life 2025; 3: 013004. 10.1103/PRXLife.3.013004

[7] Kominis IK . Phys Rev Lett 2024; 133: 263201. 10.1103/PhysRevLett.133.263201 39879051

[8] Zhao Z, Ye X, Xu S et al. Natl Sci Rev 2023; 10: nwad100. 10.1093/nsr/nwad100 37954192 PMC10632795

[9] Vinante A, Timberlake C, Budker D et al. Phys Rev Lett 2021; 127: 070801. 10.1103/PhysRevLett.127.070801 34459646

[10] Budker D, Kozlov MG. Opt Memory Neural 2023; 32: S409–14. 10.3103/S1060992X23070056

[11] Chu Y, Cai J. Phys Rev Lett 2022; 128: 200501. 10.1103/PhysRevLett.128.200501 35657873

[12] Deffner S, Campbell S. J Phys A Math Theor 2017; 50: 453001. 10.1088/1751-8121/aa86c6

